# Diabetic Cardiomyopathy: An Immunometabolic Perspective

**DOI:** 10.3389/fendo.2017.00072

**Published:** 2017-04-07

**Authors:** Paras K. Mishra, Wei Ying, Shyam Sundar Nandi, Gautam K. Bandyopadhyay, Kaushik K. Patel, Sushil K. Mahata

**Affiliations:** ^1^Department of Cellular and Integrative Physiology, University of Nebraska Medical Center, Omaha, NE, USA; ^2^Department of Anesthesiology, University of Nebraska Medical Center, Omaha, NE, USA; ^3^Department of Medicine, Metabolic Physiology and Ultrastructural Biology Laboratory, University of California San Diego, La Jolla, CA, USA; ^4^Department of Medicine, Metabolic Physiology and Ultrastructural Biology Laboratory, VA San Diego Healthcare System, San Diego, CA, USA

**Keywords:** obesity, insulin resistance, inflammation, cardiomyopathy, innate and adaptive immunity, glucose metabolism, fat metabolism, miRNA

## Abstract

The heart possesses a remarkable inherent capability to adapt itself to a wide array of genetic and extrinsic factors to maintain contractile function. Failure to sustain its compensatory responses results in cardiac dysfunction, leading to cardiomyopathy. Diabetic cardiomyopathy (DCM) is characterized by left ventricular hypertrophy and reduced diastolic function, with or without concurrent systolic dysfunction in the absence of hypertension and coronary artery disease. Changes in substrate metabolism, oxidative stress, endoplasmic reticulum stress, formation of extracellular matrix proteins, and advanced glycation end products constitute the early stage in DCM. These early events are followed by steatosis (accumulation of lipid droplets) in cardiomyocytes, which is followed by apoptosis, changes in *immune responses* with a consequent increase in fibrosis, remodeling of cardiomyocytes, and the resultant decrease in cardiac function. The heart is an omnivore, metabolically flexible, and consumes the highest amount of ATP in the body. Altered myocardial *substrate and energy metabolism* initiate the development of DCM. Diabetic hearts shift away from the utilization of glucose, rely almost completely on fatty acids (FAs) as the energy source, and become metabolically inflexible. Oxidation of FAs is metabolically inefficient as it consumes more energy. In addition to metabolic inflexibility and energy inefficiency, the diabetic heart suffers from impaired calcium handling with consequent alteration of relaxation–contraction dynamics leading to diastolic and systolic dysfunction. Sarcoplasmic reticulum (SR) plays a key role in excitation–contraction coupling as Ca^2+^ is transported into the SR by the SERCA2a (sarcoplasmic/endoplasmic reticulum calcium-ATPase 2a) during cardiac relaxation. Diabetic cardiomyocytes display decreased SERCA2a activity and leaky Ca^2+^ release channel resulting in reduced SR calcium load. The diabetic heart also suffers from marked downregulation of novel cardioprotective *microRNAs* (miRNAs) discovered recently. Since immune responses and substrate energy metabolism are critically altered in diabetes, the present review will focus on immunometabolism and miRNAs.

## Introduction

Insulin deficiency and/or resistance and elevated plasma glucose level characterize diabetes, a chronic and progressive metabolic disorder. While type 1 diabetes mellitus (T1DM) accounts for 5–10% of all cases of diabetes (1), type 2 diabetes mellitus (T2DM) accounts for the remaining ~90% of all cases of diabetes (2). As of 2015, 415 million people across the globe have diabetes mellitus (DM) (www.diabetesatlas.org), which will cost 12% of all global health expenditures (accounting for $320 billion in the USA alone) (3). The International Diabetic Federation predicts that 552 million people will suffer from diabetes by 2030. T2DM is recognized as an independent risk factor for heart failure (HF). Patients with T2DM have a greater probability of death in established HF; suffer from worse prognosis after myocardial infarction (MI) (4–8); and accounts for 5.2% of all deaths globally (9, 10). T2DM is strongly associated with obesity and sedentary lifestyle coupled with increasingly westernized diet (2, 11). Diabetic patients are also highly susceptible to diastolic dysfunction, ventricular hypertrophy, and decreased myocardial strain (12).

Rubler and colleagues initially reported diabetic cardiomyopathy (DCM) from their observation of cardiac hypertrophy on post-mortem hearts from four diabetic patients who died of HF without cardiovascular disease, which was subsequently followed by various other studies (13–16). The Strong Heart Study, the Cardiovascular Health Study, and the Framingham Study revealed cardiac hypertrophy with compromised systolic and diastolic function in DCM patients (4, 17–19). Of note, diastolic dysfunction has been reported in diabetic hearts without hypertrophy (20–22). In fact, DCM starts with diastolic dysfunction in patients with T1DM or T2DM followed by systolic dysfunction (23–27). Rodent models of T1DM including streptozotocin (STZ)-treated (28) or alloxan-treated animals (29) and T2DM models such as Goto-Kakizaki rat (30), Zucker fatty rats, Zucker diabetic fatty rats, leptin-deficient *ob/ob* mice, and leptin receptor-deficient *db/db* mice consistently show the human DCM phenotypes (31–33). Of note, STZ- and alloxan-induced diabetes is characterized by myocardial atrophy including loss of contractile proteins as opposed to cardiac hypertrophy in T2DM models (34–36). In addition, in T1DM animals, the progress of systolic dysfunction is positively correlated with the progress of the magnitude and duration of hyperglycemia (hypoinsulinemic/hyperglycemia → systolic dysfunction) (31, 35–38). By contrast, mouse models of T2DM are characterized by hyperinsulinemia, hyperglycemia (later stages), and hyperlipidemia (hyperinsulinemic/hyperglycemic → hypertrophy and diastolic dysfunction) (31, 39–41).

Autophagy is reduced in the mouse hearts of OVE26 (a transgenic model of insulinopenic diabetes) and STZ-induced diabetic mouse hearts (42–44). Metformin has been shown to prevent DCM by stimulating AMP-activated protein kinase (AMPK) activity and enhancing autophagic capacity (43).

Recently, DM is identified as a microRNA (miRNA)-related disease (45), and several diabetic complications are associated with differential expressions of various miRNAs (46). Further, miRNAs play a vital role in the regulation of metabolism (47) and since DM is a metabolic disease it is logical to examine the role of miRNAs in DM. Thus the present review will focus on altered metabolism of glucose and fatty acids (FAs) as well as immune responses in diabetes.

## Decreased Glucose Uptake and Metabolism

**Figure 1:**
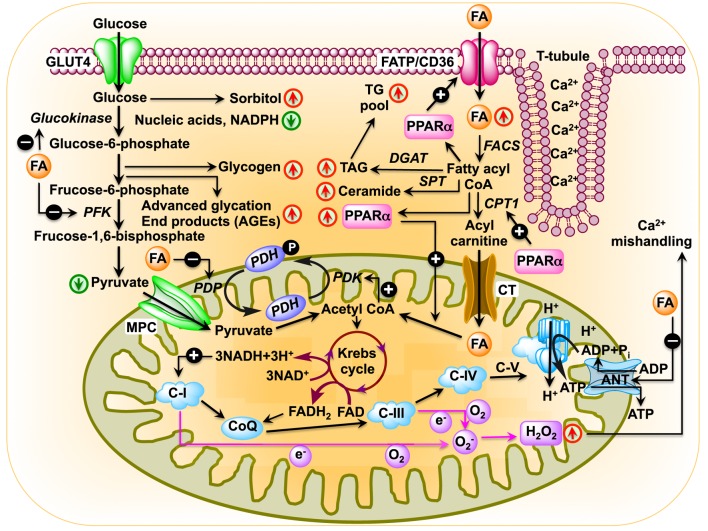
**Schematic diagram showing changes in cardiac metabolism in diabetic cardiomyopathy**. In the diabetic heart, glucose oxidation is inhibited at multiple steps: (i) uptake of glucose is inhibited by reduced expression of glucose transporter Glut4 as well as by blunted translocation of Glut4 in response to insulin (64, 65); (ii) inhibition of hexokinase activity by fatty acids (FAs) resulting in reduced conversion of glucose to glucose-6-phosphate (80); (iii) inhibition of phosphofructokinase activity by FA, leading to reduced formation of fructose-1,6-bisphosphate by fructose-6-phosphate (69); (iv) inhibition of pyruvate dehydrogenase phosphatase activity by FA resulting in reduced pyruvate dehydrogenase (PDH) activity, which leads to reduced conversion of pyruvate to acetyl CoA. In the diabetic heart, PPARα expression is activated by increased FA uptake (81, 82). Activated PPARα upregulates PDH kinase 4 enzymes, which inhibits PDH resulting in reduced production of acetyl CoA (83–85). FA transporters CD36 and FA transport protein import FAs into the cell. After import, FAs can be stored as triacylglycerol (TAG) or converted to fatty acyl CoA by fatty acyl-CoA synthetase (FACS). Carnitine palmitoyltransferase 1 (CPT1) transfers the acyl group of fatty acyl CoA to carnitine, which then shuttles into the mitochondria by carnitine translocase (CT). PPARα activates transcription of CPT1 (86). In the matrix, CPT2 reconverts the acylcarnitine back into free carnitine and fatty acyl CoA (87), which is then converted to acetyl CoA that can be used in the tricarboxylic acid to produce adenosine triphosphate by β-oxidation. Diabetes upregulates mitochondrial generation of reactive oxygen species (57, 88, 89), which affects Ca^2+^ signaling (90).

The heart consumes about 6 kg of ATP, or ~20 times its own weight, per day (48) that comes from the breakdown of fat, carbohydrate, protein, ketone bodies, or lactate. Of note, the amount of ATP in the heart is small (~10 mM, enough for only a few beats) compared with the demand (~10,000 times greater) (49). About 95% of total energy is generated from oxidative phosphorylation of FAs and glucose (50–52). A dramatic metabolic shift takes place in diabetic heart, as they rely almost completely on FAs for their energy source. As for example, 46 atoms of oxygen are required to generate 105 molecules of ATP from oxidation of 1 molecule of palmitate. By contrast, oxidation of 1 molecule of glucose utilizes 12 atoms of oxygen to generate 31 molecules of ATP. Therefore, oxidation of FAs consumes ~0.3 oxygen molecules more than glucose to generate each molecule of ATP. Thus, the diabetic heart suffers from metabolic inflexibility due to its reliance on FAs. The lack of insulin production in T1DM patients causes a dramatic decrease in cardiac glucose uptake (53, 54) where hyperglycemia increases glucose oxidation and mitochondrial generation of superoxide (55–57). Increased production of superoxide damages DNA and activates poly (ADP ribose) polymerase 1 (PARP-1) (58), which mediates inflammation and fibrosis in liver (59). PARP-1 inhibition improves cardiac function (60) and prevents hyperglycemia-induced pathological processes (61). While decreased glucose transporter type 4 (Glut4) expression in T1DM animals causes decreased glucose uptake in cardiac and skeletal muscle (62, 63), glucose uptake is impaired in T2DM hearts by decreased expression and translocation of Glut4/Glut1 (64, 65). Diabetic *db/db* mice show decreased glucose oxidation and increased reliance on FAs, indicating that insulin resistance is not responsible for metabolic switch (66–69). The high rate of FA oxidation in T2DM patients and rodents increases production of acetyl CoA and NADH, resulting in activation of pyruvate dehydrogenase kinase 4 (PDK4). PDK4 is also activated by peroxisome proliferator-activated receptor alpha (PPARα), which is overexpressed in diabetic rodents (70–73). Activated PDK4 inhibits pyruvate dehydrogenase complex, thereby preventing oxidation of pyruvate (74, 75) (Figure 1). In addition, increased accumulation of FAs and their derivatives fatty acyl CoA, diacylglycerol, and ceramide activate protein kinase C, c-Jun N-terminal kinases, mammalian target of rapamycin, and inhibitor of κB kinase β with consequent decrease in insulin signaling (76–79).

## Increased Formation of Advanced Glycation end Products (AGEs)

In the diabetic heart, glucose forms covalent adducts with the plasma proteins through a non-enzymatic reaction between the free amino groups of proteins and carbonyl groups of reducing sugars, resulting in the formation of stable glycosylation products by Amadori rearrangement, which is called glycation (91–94). Glycated proteins undergo a series of oxidation, dehydration, and cyclization reactions to form long-lived AGEs (95, 96). Both AGE and its receptor RAGE are overexpressed in diabetes (97) leading to the generation of reactive oxygen species (ROS) and subsequent activation of RAS–MAP kinase pathway (98). Activation of RAS–MAPK pathway in turn activates NF-κB pathway resulting in decreases in contractile proteins such as α-actin and myosin ATPase activity (35, 36) and shifts in myosin heavy chain isoforms from α to β with consequent development of decreased systolic tension (36–38, 97, 99). In diabetes, increased serum levels of AGEs show positive correlation with ventricular isovolumetric relaxation time, arterial stiffness, and carotid intimal thickness (100–102). Treatment of diabetic animals with aminoguanidine (an inhibitor of AGE formation) (103, 104) or with alagebrium (ALT-711; disrupts AGE cross-link) (105) restored LV function and reduced myocardial collagen, highlighting the importance of AGE in cardiac dysfunction. AGEs also impair collagen degradation by matrix metalloproteinases (MMPs), such as MMP2, resulting in increased fibrosis (106, 107). Fibrosis increases myocardial stiffness and impairs diastolic function (104). In T1DM heart, AGEs also induce cross-linking of SERCA2a pump, thereby attenuating sarcoplasmic reticulum (SR) Ca^2+^ reuptake (108, 109) with consequent attenuation of the maximum and minimum rate of pressure change in the ventricle and LV developed pressure (108). Of note, the type 2 ryanodine receptor-dependent Ca^2+^ release not only plays critical roles for excitation–contraction coupling in cardiomyocytes but plays crucial roles in the regulation of insulin secretion and glucose homeostasis (110, 111). Genetic ablation of the RAGE gene improves hemodynamic dysfunction, thereby providing AGE/RAGE pathway as a potential therapeutic target to alleviate cardiac dysfunction in diabetes.

## Increased FA Uptake and Metabolism

The heart has a limited capacity for *de novo* synthesis of FAs. Therefore, it relies heavily on the circulating FAs (112). FAs translocate from blood to cardiomyocytes using three FA transporters: cluster of differentiation 36 (CD36), FA transport protein 1, and the plasma membrane form of FA-binding protein (113–116). Increased PPARα expression in diabetic hearts (70–73, 117) augments transcription of FA transporters. About 75% of the translocated FAs are transferred to mitochondria for the generation of ATP and the rest are converted to triacylglycerol (TAG) for future use (118). Translocated FAs are activated by esterification to fatty acyl CoA by the action of cytosolic fatty acyl-CoA synthetase (FACS). Carnitine palmitoyltransferase 1 (CPT1) exchanges the CoA moiety of fatty acyl CoA for carnitine resulting in the formation of acylcarnitine. Acylcarnitine is transported across the inner mitochondrial membrane into the matrix by carnitine–acylcarnitine translocase. PPARα augments transcription of CPT1 (119, 120). In the matrix, CPT2 reconverts the acylcarnitine back into free carnitine and fatty acyl CoA. PPARα increases transcription of CPT2 (120). Fatty acyl CoA is then converted to acetyl CoA for β-oxidation and generation of ATP. PPARα increases conversion of fatty acyl CoA in the mitochondrial matrix to acetyl CoA. Thus, PPARα plays critical roles in metabolic reprograming in diabetic hearts.

Since the diabetic heart relies on FAs for ATP generation, it consumes ~30% more oxygen compared with non-diabetic heart to generate similar levels of ATP (87, 121) and generate the same or the reduced amounts of contractile force (41). This disproportionate use of FAs also alters cellular ATP shuttling as long-chain acyl CoA derivatives inhibit the adenine nucleotide translocator for the transport of ATP from mitochondria to the cytosol (122–124), eventuating in inefficient delivery of ATP to myofibrils that affects cardiac contractility.

## Inflammation, Innate, and Adaptive Immune Responses

**Figure 2:**
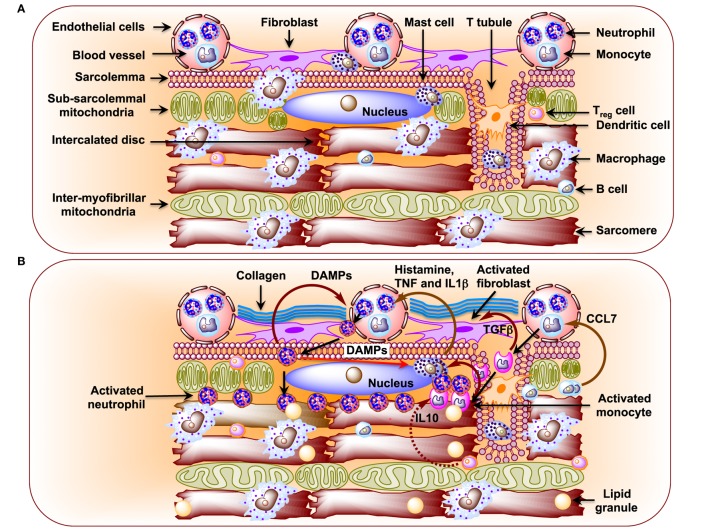
**(A)** Schematic diagram showing immune cells in the healthy heart. Macrophages are the preponderant immune cells in the resting heart and reside primarily surrounding endothelial cells and also in the interstitium among cardiomyocytes (141–143). The less preponderant immune cells include the following: mast cells, dendritic cells, B cells, and regulatory T (T_Reg_) cells (142, 144, 147). Neutrophils and monocytes, in general, are not detected in the resting heart. **(B)** Schematic diagram showing infiltration of neutrophils and monocytes from the circulation and their effects on resident immune cells in the diabetic cardiomyopathy (DCM) heart. In DCM, activated B cells release CCL7 that activates blood monocytes (146). Upon infiltration activated monocytes stimulate mast cells to release histamine, TNF, and interleukin 1β (IL1β), which activate neutrophils in circulation (144, 174). Activated neutrophils infiltrate heart and activate mast cells through damage-associated molecular patterns as well as blood neutrophils. Activated monocytes secrete TGFβ, which activates fibroblasts to induce formation of collagen.

Metabolic disturbances induce subcellular low-grade inflammation in the heart (125). Inflammation is a key pathogenic feature of lipid excess and diabetes. The innate immune system comprising of neutrophils, dendritic cells, macrophages, mast cells, and eosinophils also induces chronic metabolic inflammation (126, 127). Myocardial inflammation is implicated in the development of DCM (128–131). Nuclear factor kappa-light-chain-enhancer of activated B cells (NF-κB), a primary regulator of inflammatory responses, is activated in the heart upon exposure to FAs or glucose (132, 133). NF-κB induces not only the expression of pro-inflammatory cytokines, such as tumor necrosis factor alpha (TNFα), interleukin 6 (IL6), pro-IL1β, and pro-IL18, but it also induces the expression of NLR family pyrin domain-containing 3 (NLRP3) inflammasome (134). Activated RAGE also triggers an inflammatory response by heterodimerizing with TLR-4 leading to the production of pro-IL1β, Pro-IL18, and NLRP3 (135). Activated NLRP3 inflammasome activates caspase-1 and mediates the processing and release of pro-inflammatory cytokines IL1β and IL18 resulting in inflammatory cell infiltration and amplification of the inflammatory response (125, 136–138). Likewise, depletion of NLRP3 attenuates inflammation and cardiomyopathy in T2DM rats (137). Of note, activated inflammasomes play critical roles in the pathogenesis of HF (139). Resident immune cells in the resting heart include the following: macrophages, residing near endothelial cells or within the interstitial space (140–143); mast cells that are responsible for early triggers of immune responses (144); a small number of adaptive immune cells: B cells and regulatory T (T_Reg_) cell subsets (142, 145, 146); and dendritic cells that test sample antigens (142, 147) (Figure 2A). The differential expression of major histocompatibility complex (MHC) class II and CC chemokine receptor 2 (CCR2) distinguishes three different subsets of cardiac macrophages: MHC class II^high^ (CCR2^−^), MHC class II^low^ (CCR2^−^), and CCR2^+^ macrophages. The first two are the preponderant macrophages in the heart, derived from embryogenic progenitors and renewed through *in situ* proliferation, rather than through monocyte input. By contrast, CCR2^+^ macrophages derive from and replenished by circulating blood monocytes, which comprise of Ly6C^high^ and Ly6C^low^ (148–150). Studies in *Ccr2* knockout mice (lacking circulating monocytes) reveal increased cardiac pathology (151, 152). The loss of Ly6C^high^ monocytes prevents hypertension-induced cardiac fibrosis and improves cardiac function after MI (141, 153, 154). Monnerat et al. suggest that diabetes enhances IL1β production from cardiac MHC II^high^ pro-inflammatory macrophages through activation of TLR2–NLRP3 inflammasome axis (155). The increased level of IL1β leads to a reduction in potassium current and an increase in calcium sparks in cardiomyocytes, which cause cardiac arrhythmias (156). By contrast, M2-like macrophages (CD206^+^F4/80^+^CD11b^+^) exert profound functions on tissue repair in heart depending on IL4 secretion (156). Recent studies implicate TNFβ producing B cells as a major contributor to myocardial fibrosis (153, 157). Antigen and cytokine stimulation are known to differentiate naive T cells into distinct T cell subpopulations that include T helper cells and CD4^+^CD25^+^FOXP3^+^ T_Reg_ cells (158). T_Reg_ cells comprise a subset of CD4^+^ lymphocytes that suppress activation, proliferation, and effector responses of both innate and adaptive immune cells (159–161). As opposed to B cells, depletion of T_Reg_ cells aggravates myocardial fibrosis and adoptive transfer of exogenous T_Reg_ cells into these mice attenuates the extent of myocardial fibrosis (158). The following pro-inflammatory changes with oxidative stress and decreased cardiac function were detected in STZ-induced rat model of T1DM (162): (i) significant increases in myocardial intercellular adhesion molecule 1 and vascular cell adhesion molecule 1, (ii) increased expression of beta2-leukotrienes-integrins^+^ (CD18^+^, CD11a^+^, CD11b^+^), (iii) increased expression of TNFα, and (iv) IL1β (Figure 2B). Treatment of STZ-induced DCM rats with irbesartan (AT-1 receptor antagonist) has been reported to improve cardiac functions by attenuating cardiac inflammation (IL1β, TNFα, and TGFβ) and restoration of MMP activity with consequent decrease in fibrosis (107). Similar results were reported after neutralization of TNFα (163) or genetic deletion of neurokinin receptor B (164) in rodent models of T1DM. Subsequently, several studies confirmed the pro-inflammatory phenotypes in diabetic rodent heart (165–167). As opposed to metabolic responses, immune responses in T1DM and T2DM are comparable as both of them show consistent activation of pro-inflammatory transcription factor NF-κB. Cytokines (i) increase formation of peroxynitrite, which play critical roles in cardiac dysfunction (168), (ii) exert direct effects on the function of SR as well as on the regulation of SR calcium ATPase expression (168, 169), and (iii) increase fibrosis (170). Treatment of rats and humans with statins (171), renin angiotensin aldosterone system (RAAS) inhibitors (107), metformin (172), and thiazolidinediones (173) reduces inflammation in the heart and improve cardiac function.

## miRNA in Diabetic Hearts

MicroRNAs are highly conserved endogenous small non-coding RNAs, ~22 nucleotides in length, that regulate gene expression by binding to partially complementary sequences of mRNA (175). The failing hearts consistently show chronic immune activation and aberrant miRNA expression (176). Thus, miR-155 plays an important role in the mammalian immune systems as well as during HF and is abundantly expressed in T-cells, B-cells, and monocytes (177–180). miRNAs are also differentially expressed during HF (181). STZ-induced diabetic heart expresses higher levels of miR-195 and silencing of miR-195 reduces DCM (182). Likewise, miR-141 is increased in diabetic heart and affects mitochondrial function and ATP generation (183). Palmitate-stimulated neonatal rat cardiomyocytes (NRCs) and diet-induced obese (DIO) mouse heart also showed increased expression of miR-451, which decreases LKB1/AMPK signaling (184). Expression of miR-133a reduces Glut4 expression with consequent decrease in insulin-mediated glucose uptake in NRCs (185). While overexpression of miR-223 in NRCs significantly increased glucose uptake by increasing total Glut4 level and its translocation, inhibition of miR-223 in the heart resulted in a significant decrease in Glut4 expression (186). In contrast to the findings in NRCs, expression of miR-133a is decreased in the hearts of diabetic mice and is associated with increased fibrosis. Of note, overexpression of miR-133a in the heart attenuates cardiac fibrosis (187). Murine miR-322 has recently been shown to provide cardioprotection against consequences of hyperinsulinemia and hyperlipidemia (188). In Ins+/– Akita mice, a model for T1DM, the majority of miRNAs are downregulated in the heart (189), including miR-133a which regulates contractility of the diabetic heart (190). Even after treatment with insulin, which normalizes blood glucose levels, there are several miRNAs that remain differentially regulated in the diabetic heart, and they can potentially contribute to pathological remodeling of the diabetic heart (191). These miRNAs could be a potential target for developing a novel therapeutic strategy for the treatment of diabetic HF.

Diabetes mellitus is a metabolic disease, and miRNAs play a crucial role in the regulation of metabolism (47). Increased levels of plasma cholesterol and triglyceride are common in diabetes, and liver specific ablation of miR-122, the most abundant miRNA in the liver, reduces plasma cholesterol and triglyceride levels (192, 193). The intracellular cholesterol and FA homeostasis are controlled by miR-33a and miR-33b, which target genes involved in cholesterol export including adenosine triphosphate-binding cassette transporters (194–196). Endogenous inhibition or knockout of miR-33 leads to increased plasma high-density lipid levels (194–197). MiR-223 controls the expression of *Glut4* gene in cardiomyocytes, and miR-223 is upregulated while Glut4 is downregulated in human diabetic hearts (186). The switch of glycolysis to FA oxidation is regulated by PPARδ, which is regulated by the miR-199/miR-214 cluster. The miR-199/miR-214 cluster downregulates PPARδ and impairs FA oxidation (198). ROS stimulates apoptosis by mitochondrial cytochrome *c* release and ceramide generation (199). In rat cardiomyocytes, high glucose upregulates miR-34a and miR-1 that reduces the levels of B-cell lymphoma 2 (*Bcl-2*) and insulin-like growth factor 1 (*Igf-1*) genes, respectively, and induces apoptosis (200, 201). Recently, Kuwabara et al. has elegantly shown that miR-451 plays a key role in exacerbating lipotoxicity in cardiac myocytes and high-fat diet-induced cardiac hypertrophy in mice through suppression of the LKB1/AMPK pathway (184). MiR-133a, the most abundant miRNA in the heart, is downregulated in the diabetic mice heart with consequent induction of cardiac hypertrophy (202) and fibrosis (187). Lack of miR-133a also causes contractile dysfunction in the diabetic mice heart (190). These changes cause diastolic dysfunction, which if untreated leads to potential systolic dysfunction (28).

## Conclusion and Future Perspectives

Cardiovascular disease has remained the leading cause of mortality and morbidity in individuals with diabetes. DCM is emerging as an increasing health concern with the epidemic rise in DM worldwide. Animal studies have clearly shown that glycemic control at an early stage prevents the development of DCM, and that certain anti-diabetic drugs exert anti-remodeling effects. While a large body of epidemiological evidence (50,000 T2DM patients) indicate a positive correlation between blood glucose level and/or HbA1c and the risk of HF (203–205), a meta-analysis of randomized controlled trials (37,229 patients) showed no effect of intensive glycemic control on the risk of HF in T2DM patients (206).

Therapeutic approach for DCM depends mainly on (i) glycemic control, (ii) glucose-lowering drug administration, (iii) improvement of autophagy, and (iv) an active life style. Earliest detection, helped by current research on miRNAs, will enhance therapeutic efficacy. The current burst of scientific evidence for the potential use of circulating miRNAs as biomarkers for cardiomyopathy is generating hopes that someday soon detection of specific miRNAs in biofluids of patients will help early treatment of both diabetes and cardiomyopathy.

## Author Contributions

SM conceived the idea, wrote the immunometabolism part of the manuscript, and made the schematic diagrams. PM wrote the microRNA part of the manuscript and contributed in correcting the final draft of the manuscript. SN contributed to drafting and correcting of the final version of the manuscript. WY, KP, and GB participated in discussion and reviewed/edited the manuscript.

## Conflict of Interest Statement

The authors declare that the research was conducted in the absence of any commercial or financial relationships that could be construed as a potential conflict of interest.
